# Diagnostic Yield of Fusion-Guided and Randomized Biopsies in Prostate Cancer: Evidence for an Integrated Approach

**DOI:** 10.3390/healthcare13172214

**Published:** 2025-09-04

**Authors:** Osama Salloum, Iulian-Alexandru Taciuc, Alexandru Dick, Costin Petcu, Costin Gingu, Nicoleta Sanda, Andreea Nicoleta Marinescu, Crenguta Serboiu, Adrian Costache

**Affiliations:** 1 Pathology Department, Carol Davila University of Medicine and Pharmacy, 050474 Bucharest, Romania; os.salloum@gmail.com (O.S.); adriancostacheeco@yahoo.com (A.C.); 2 Nuclear Medicine Department, Carol Davila University of Medicine and Pharmacy, 050474 Bucharest, Romania; alexandertaciuc@gmail.com; 3 Urology Department, Carol Davila University of Medicine and Pharmacy, 050474 Bucharest, Romania; alextrooper@gmail.com (A.D.); petcu.costin@yahoo.com (C.P.); cgingu@gmail.com (C.G.); 4 General Surgery Department, Carol Davila University of Medicine and Pharmacy, 050474 Bucharest, Romania; 5 Imaging Department, Carol Davila University of Medicine and Pharmacy, 050474 Bucharest, Romania; andreea_marinescu2003@yahoo.com; 6 Molecular Biology and Histology Department, Carol Davila University of Medicine and Pharmacy, 050474 Bucharest, Romania; crengutas@yahoo.com

**Keywords:** fusion biopsy, multivariate analysis, PI-RADS, prostate cancer, systematic biopsy

## Abstract

**Background/Objectives:** Improving prostate cancer (PCa) detection remains a key clinical goal. While multiparametric MRI (mp-MRI) fusion-guided biopsy has shown advantages over systematic randomized biopsy, variability persists across studies. This study aimed to compare detection rates between fusion-guided and randomized biopsy techniques and assess the combined predictive value of clinical risk factors. **Methods:** We retrospectively analyzed 138 male patients aged 50–82 years with PSA (prostate-specific antigen) < 25 ng/mL, undergoing both mp-MRI fusion-guided and systematic randomized biopsies. PI-RADS v2.1 was used for lesion assessment. The patient data included PSA, prostate volume, PI-RADS score, and age. Multicollinearity was evaluated, and a multivariate logistic regression model was developed. ROC analysis assessed predictive performance. **Results:** Fusion-guided biopsy detected cancer in 68.1% (95% CI: 60.3–75.9%) of cases, randomized biopsy in 76.1% (95% CI: 68.9–83.2%), and the combined approach in 88.4% (95% CI: 83.1–93.7%). McNemar’s test confirmed a significant improvement when combining both methods (*p* < 0.001). PSA exhibited the strongest individual predictive power (AUC = 0.782, 95% CI: ~0.70–0.86), followed by prostate volume (AUC = 0.631, 95% CI: ~0.53–0.73), PI-RADS score (AUC = 0.619, 95% CI: ~0.51–0.72), and age (AUC = 0.572, 95% CI: ~0.46–0.68). The multivariate model achieved an AUC of 0.751 (95% CI: ~0.66–0.83) and an accuracy of 89.6%. **Conclusions:** Combining fusion-guided and randomized biopsy techniques enhances prostate cancer detection compared with either method alone. PSA, prostate volume, PI-RADS score, and age contribute independently to risk prediction. Future studies will aim to refine stratification models and explore familial cancer risk factors.

## 1. Introduction

In developed countries, prostate cancer (PCa) continues to be one of the most prevalent malignancies among men, particularly those in middle age. With an annual incidence of approximately 190,000 new cases in North America, there is a persistent focus on early-stage detection of the disease [1]. In Europe, the European Cancer Information System (ECIS, official EU source) reported 335,514 new cases of prostate cancer and 69,945 deaths in 2020. The highest incidence rates occurred in Sweden, Estonia, and Ireland, ranging from 221 to 250 cases per 100,000 men. Prostate cancer-specific mortality was highest in Estonia and Slovakia, with 67 to 78 deaths per 100,000 men. In Romania, the Joint Research Centre reported incidence rates of 101 to 131 per 100,000 men and mortality rates of 33 to 45 per 100,000. Consequently, various screening techniques have demonstrated their effectiveness, including prostate-specific antigen (PSA) testing and digital rectal examinations [2].

To detect low-volume prostate cancer, suspicion arising from PSA levels and digital rectal examinations is followed by an ultrasound-guided prostate biopsy. The transrectal ultrasound (TRUS)-guided prostate biopsy, typically performed with 12 cores, has been regarded as the gold-standard procedure for prostate cancer detection [3,4]. Several studies have also shown that prostate cancer detection rates tend to decrease as prostate volume increases, particularly when the number of biopsy cores is limited [5]. In recent large-scale studies involving over 30,000 patients with nocturia, digital rectal examination was found to be only modestly accurate in approximating prostate volume [6]. Nonetheless, it remains a valuable component of the diagnostic process when clinical suspicion is raised, as each evaluation method contributes uniquely.

Serum PSA measurement is one of the most important biomarkers in prostate cancer detection; however, its widespread use has also led to an increase in the diagnosis of clinically insignificant tumors worldwide. In response to concerns about overdiagnosis and overtreatment, recent research has supported risk-adapted screening strategies (such as establishing a baseline PSA value at age 45) to stratify risk and determine appropriate testing intervals. Additionally, active surveillance and imaging tools such as mp-MRI are increasingly used to guide decisions and reduce unnecessary interventions [7].

The mp-MRI is a method that includes both anatomical (T1- and T2-weighted) and functional (diffusion- and dynamic contrast-enhanced) sequences [8]. Besides diagnostic accuracy, it can also reveal other incidental findings such as bone metastases related to PCa [9]. The fusion between the real-time TRUS-guided biopsy with mp-MRI allowed targeting the higher risk zones, thus decreasing the number of punctures [4]. There are plenty of studies demonstrating that the fusion biopsy increases the chance of detecting PCa [10,11]. One of the most valuable tools provided by mp-MRI is the Prostate Imaging Reporting and Data System (PI-RADS). The PI-RADS divides the prostate into multiple regions, typically between 16 and 27, and assigns a score to individual lesions based on their likelihood of being clinically significant prostate cancer. This standardized approach enhances the consistency of lesion characterization, aiding risk stratification and guiding biopsy decisions [12]. Since September 2019, PI-RADS version 2.1 has been available. Key updates in this revised version include the reclassification of certain imaging features. Notably, encapsulated, round nodules are now classified as PI-RADS 1, reflecting their benign appearance. Additionally, transition zone lesions with a T2-weighted (T2W) score of 2 remain PI-RADS 2 if the dynamic contrast-enhanced (DCE)/Apparent Diffusion Coefficient (ADC) score is equal to or less than 3, but are upgraded to PI-RADS 3 if the DCE/ADC score is equal to or greater than 4 [13].

False-negative results in guided biopsy are not uncommon, raising concerns about missed diagnoses, particularly when multiparametric MRI (mp-MRI) identifies a potential lesion. Conversely, mp-MRI can also produce false-positive findings, detecting lesions that mimic prostate cancer (PCa mimics), which may lead to unnecessary biopsies [14].

Serum PSA measurement is one of the most important biomarkers in prostate cancer detection; however, its widespread use has also led to an increase in the diagnosis of clinically insignificant tumors worldwide [1].

This study aims to compare randomized prostate biopsy with fusion-guided biopsy in patients with low PSA values, both of which were performed on all patients, thereby addressing a key question in the literature: which method is superior? We aim to bridge this gap by demonstrating that the answer is both combined.

This retrospective study assessed and compared the diagnostic performance of multiparametric MRI-guided fusion biopsy and systematic randomized biopsy for detecting prostate cancer in 138 patients with PSA levels below 25 ng/mL. By incorporating clinical risk factors—including PSA, prostate volume, PI-RADS score, and age—into a multivariate logistic regression model, this study provides a robust evaluation of their individual and combined predictive values. The structured methodology and comprehensive reporting align with current research standards, ensuring suitability for inclusion in future systematic reviews and meta-analyses on biopsy accuracy and prostate cancer diagnostic strategies.

## 2. Materials and Methods

### 2.1. Study Design and Ethical Approval

A retrospective analysis was conducted on a database of 891 patients managed within a single healthcare unit between June 2022 and December 2024. This study was performed at a private tertiary unit and was approved by the ethics committee (No. 17/2024). Data were collected between December 2024 and February 2025.

### 2.2. Participants and Sampling

In total, 891 male patients who underwent prostate evaluation were initially reviewed.

Inclusion criteria were as follows: availability of pre-biopsy prostate-specific antigen (PSA) levels; completion of multiparametric magnetic resonance imaging (mp-MRI); availability of pathological results; informed consent to receive the institutional prostate biopsy protocol (described in subsequent sections); and performance of both fusion-guided and systematic random biopsies on the same day, within a single procedure.

**Figure 1:**
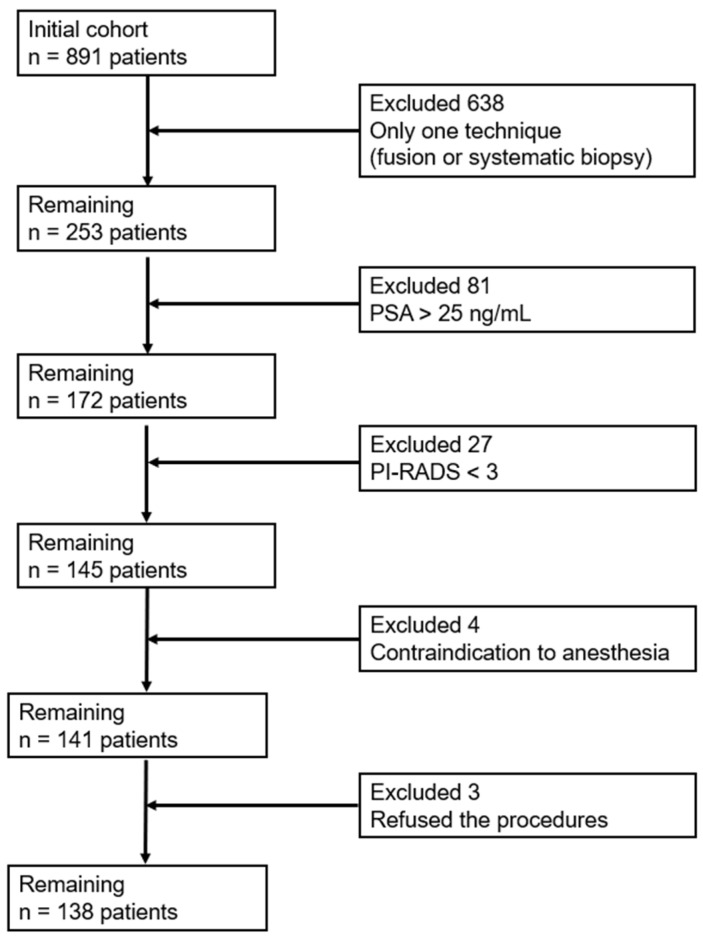
PRISMA Flow Sheet diagram.

Exclusion criteria included refusal to undergo the procedure, contraindications to anesthesia, a PI-RADS score < 3, PSA levels > 25 ng/mL, and anteriorly located lesions. After applying these criteria, 138 patients were eligible for final analysis (Figure 1).

### 2.3. Patient Preparation

All patients received fluoroquinolone antibiotics prior to biopsy to exclude inflammatory causes of PSA elevation (all included patients had PSA < 25 ng/mL). PSA levels were reassessed after completion of antibiotic treatment, and the post-treatment values were recorded for analysis. All patients were informed about the benefits and risks of the procedure and signed an informed consent form. Prior to the procedure, each patient underwent a pre-anesthetic consultation to assess eligibility for intravenous sedation. On the day of the biopsy, the patients received a micro-enema and a prophylactic dose of antibiotics.

### 2.4. Imaging and PI-RADS Scoring

The mp-MRI was performed using a 3 Tesla Siemens Magnetom Skyra system (Siemens Medical Solutions USA, Inc., Malvern, PA, USA). The imaging protocol included triplane T2W, DWI, and dynamic contrast-enhanced (DCE) pelvic sequences, as well as post-contrast abdominopelvic imaging. All scans were interpreted by two experienced radiologists, and lesions were evaluated according to the PI-RADS version 2.1 criteria. The radiologists were granted access to corresponding blood test results for diagnostic correlation.

### 2.5. Biopsy Procedure

Biopsies were obtained via the transrectal approach under local anesthesia and supplemented with intravenous sedation. Fusion-guided biopsy was conducted using a BK 5000 ultrasound system (Mileparken 34, 2730 Herlev, Denmark) equipped with a 7 MHz endorectal transducer (Mileparken 34, Herlev, Denmark). Tissue samples were collected using a Bard Magnum Gun (Bard, Franklin Lakes, NJ, USA) (250 mm, 18G needle). For the fusion-guided component, two to six cores were obtained from each identified lesion, depending on the lesion’s volume. The protocol required at least two core samples from smaller lesions, which were obtained from different regions, and up to six cores for lesions larger than 125 cc.

Fusion-guided biopsy was immediately followed by a systematic randomized biopsy, in which 12 cores were collected according to the standard sextant protocol. The biopsies were performed under local anesthesia combined with intravenous sedation, administered following a pre-anesthetic consultation.

Each procedure was carried out by a team consisting of a urologist, an anesthesiologist, and a surgical nurse.

### 2.6. Histopathological Analysis

Two experienced pathologists examined all biopsy specimens according to standardized laboratory protocols, with access to blood test results and PI-RADS scores. Diagnoses included Gleason scores ranging from 6 to 9, as well as benign prostatic hyperplasia (BPH), high-grade prostatic intraepithelial neoplasia (HGPIN), atypical glands suspicious for malignancy (ATYP), and a rare case of amyloidosis. A biopsy was classified as positive if at least one core from either method contained malignant cells.

### 2.7. Data Collection

We extracted the following data from each patient’s record: age, most recent pre-biopsy PSA level, prostate volume, PI-RADS score, and biopsy outcomes from both fusion-guided and systematic approaches.

**Figure 2:**
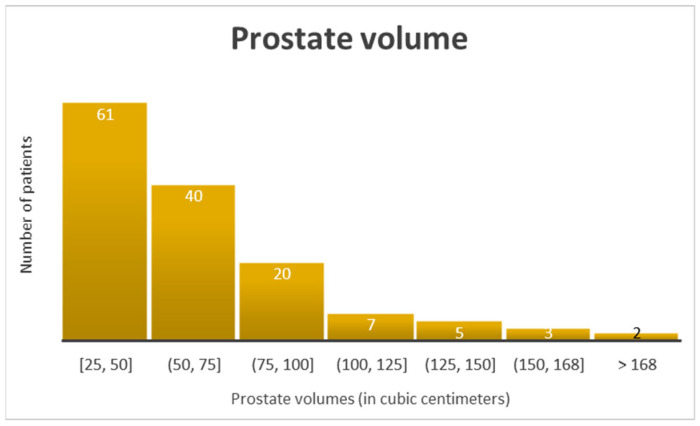
Distribution of patients according to prostate volume (cc).

To illustrate the heterogeneity of the dataset, all selected patients were male, aged 50–82 years, with an interquartile range indicating a diverse age distribution. PSA levels were below 25 ng/mL, and all patients had PI-RADS scores ≥ 3, with most classified as PI-RADS 4 or 5. None of the patients had a prior history of prostate biopsy. Prostate volume showed wide variability, ranging from 25 to 214 cc (Figure 2). For improved interpretability, the patients were stratified according to key clinical risk factors. PSA levels were grouped into three categories: low (<6 ng/mL), intermediate (6–11 ng/mL), and elevated (>11 ng/mL). Prostate volume was stratified into 25 cc intervals to capture granular variation across the cohort. The PI-RADS scores were dichotomized into PI-RADS 3 (equivocal) and PI-RADS 4–5 (likely or very likely to represent clinically significant cancer). Age was categorized into four groups (45–55, 56–65, 66–75, and ≥76 years) consistent with the distribution of the study population. These variables were incorporated into a multivariate logistic regression model to evaluate their combined predictive value for clinically significant prostate cancer. The single-center design and selective inclusion criteria—restricting participants to those with PI-RADS scores ≥ 3 and no prior biopsy history—may introduce selection bias and limit external validity. These factors should be considered when interpreting the generalizability of the results to broader, more heterogeneous populations.

### 2.8. Statistical Analysis

A database was created using Microsoft Excel 2019. Although the data were managed in this database, we implemented quality control procedures to ensure data integrity. These measures included double-checking entries, applying data validation rules (e.g., restricting inputs to predefined values and using dropdown lists for repetitive variables), and limiting database access.

Statistical analysis was performed using JASP 0.19.3. A *p*-value of <0.05 was considered statistically significant. We applied logistic regression to assess the predictive value of PSA, prostate volume, PI-RADS, and age. Receiver Operating Characteristic (ROC) analysis evaluated diagnostic performance. A *p*-value of <0.05 was considered statistically significant.

## 3. Results

**Figure 3:**
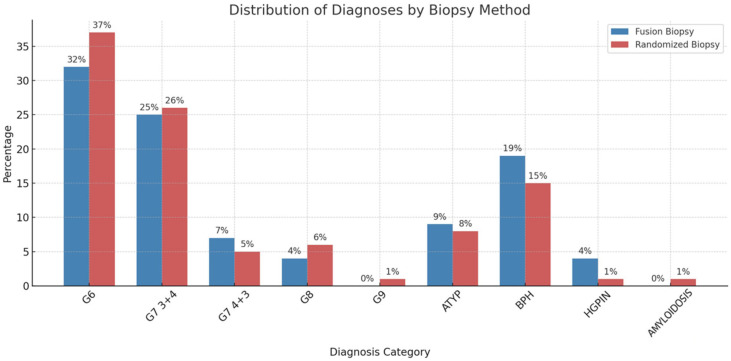
Distribution of histopathological findings in percentage detected through fusion-guided biopsy and randomized biopsy.

The biopsy results are summarized in Table 1 and Figure 3. The histopathological findings detected through fusion-guided biopsy included 44 patients with G6(3+3), 34 patients with G7(3+4), 10 patients with G7(4+3), five patients with G8(4+4), 13 patients with ATYP, six patients with HGPIN, and 26 patients with BPH. On the other hand, at randomized biopsy, the histopathological findings revealed 49 patients with G6(3+3), 35 patients with G7(3+4), six patients with G7(4+3), eight patients with G8(4+4), one patient with G9 (5+4), 10 patients with ATYP, 20 patients with BPH, two patients with HGPIN, and one rare case of amyloidosis. Thus, of the 138 patients, 94 patients were positive for PC at fusion biopsy (F+), 105 patients were positive at randomized biopsy (R+), 16 patients were positive at fusion biopsy but negative at randomized biopsy, and 28 patients were positive at randomized biopsy and negative at fusion biopsy. A total of 16 patients were negative at both fusion and randomized biopsies. In our dataset, we identified cases in which one biopsy technique diagnosed benign prostatic hyperplasia (BPH) while the other detected clinically significant prostate cancer (Gleason score 6 or 7 [3+4]). This discrepancy likely reflects intrinsic differences between the two methods. Systematic biopsy samples a broader distribution of the prostate, increasing the chance of detecting multifocal disease that targeted imaging might miss. In contrast, fusion-guided biopsy uses mp-MRI to directly visualize suspicious lesions, allowing more precise sampling of areas most likely to harbor malignancy. These findings highlight the complementary nature of both techniques and support their combined use to improve diagnostic accuracy.

Since we have aggregate data, we first ran McNemar’s test to compare the diagnostic value of fusion versus randomized biopsies. The statistic test was 2.75 with a *p*-value of 0.097. Since the *p*-value is >0.05, the difference between the fusion-only positive biopsies and randomized-only positive cases is not statistically significant. This result suggests that based on our data, there is no strong evidence that one technique alone detects more PC than the other.

When evaluating detection rates, fusion biopsy alone identified 68.1% of cases (95% CI: 60.3–75.9%), while randomized biopsy alone identified 76.1% (95% CI: 68.9–83.2%). However, combining both techniques (fusion positives ∪ randomized positives) increased the overall detection rate to 88.4% (95% CI: 83.1–93.7%) (122 patients). This represents an absolute gain of 20.3% (95% CI: 10.9–29.7%) compared with fusion alone and 12.3% (95% CI: 3.4–21.2%) compared with randomized biopsy alone. All discordant biopsy cases—those detected using fusion only (F+/R−) or via systematic biopsy only (R+/F−)—were confirmed as clinically significant prostate cancers (Gleason scores 6 or 7 [3+4]), highlighting the complementary value of both biopsy approaches for accurate cancer detection. To evaluate the statistical significance of this improvement, McNemar’s test was applied again since it is specifically designed to detect significant differences in matched-pair data such as ours. The comparison between fusion biopsy alone and the combined approach (Table 2) yielded a *p*-value of 3.35 × 10^−7^, while the comparison between randomized biopsy alone and the combined approach (Table 2) produced a *p*-value of 0.0001. These results indicate that the combined approach significantly outperforms each technique used in isolation, supporting its use for improving the diagnostic yield in prostate cancer detection.

We also analyzed the correlation between the PSA values, prostate volumes, age, and PI-RADS score. Multicollinearity diagnostics were performed for the independent variables (age, PSA, PI-RADS score, and prostate volume). All predictors demonstrated acceptable tolerance values (>0.78) and low Variance Inflation Factor values (VIF < 1.3), indicating no significant multicollinearity and supporting their inclusion in the multivariate regression model (Table 3). The resulting multivariate model demonstrated strong discriminative power, with an overall accuracy of 89.6% and an area under the ROC curve (AUC) of 0.751 (95% CI: ~0.66–0.83). To further assess the individual predictive performance of each covariate, separate ROC curve analyses were conducted. Among the tested variables, PSA level demonstrated the highest discriminative power, with an AUC of 0.782 (95% CI: ~0.70–0.86), suggesting a strong ability to differentiate between patients with and without clinically significant prostate cancer. Prostate volume showed a moderate predictive value, with an AUC of 0.631 (95% CI: ~0.53–0.73), indicating some association with cancer detection but with limited standalone accuracy. Despite its role as a structured imaging classification system, the PI-RADS score yielded an AUC of 0.619 (95% CI: ~0.51–0.72), indicating only modest performance when applied in isolation; this discrepancy with prior research is addressed in Section 4.4. Finally, age had the lowest individual predictive performance, with an AUC of 0.572 (95% CI: ~0.46–0.68), suggesting that while age remains an important clinical factor, it does not independently offer strong discriminative power in isolation. Due to the relatively small dataset, we deemed it appropriate to apply the Hanley & McNeil formula for calculating the confidence intervals of the AUC values. These findings underscore the importance of combining multiple parameters in a multivariate model, where their collective contribution leads to a more robust and accurate prediction—as reflected in the overall model AUC of 0.751 and an accuracy of 89.6%.

## 4. Discussion

### 4.1. General Discussion

Although several of the clinical predictors used in this model, such as PSA level, age, prostate volume, and PI-RADS score, are known to be individually associated with prostate cancer risk, multicollinearity diagnostics confirmed that these variables contributed independently to the model. Specifically, tolerance values exceeded 0.78 and VIF values remained below 1.3 for all predictors, indicating no significant multicollinearity. This finding suggests that despite some expected clinical overlap (e.g., PSA levels may rise with age or prostate enlargement), each variable retained a distinct predictive value in identifying clinically significant prostate cancer. This enhances the interpretability and reliability of the regression model and supports the continued use of a multifactorial approach in risk stratification. From a clinical perspective, these findings hold importance for several reasons. First, they validate the use of a multifactorial approach in risk stratification by demonstrating that clinicians can combine commonly used parameters without compromising model integrity. This validation encourages the development of clinically actionable tools that integrate multiple inputs and guide biopsy decisions more accurately than reliance on any single factor. Second, each predictor contributes independently, which strengthens confidence in applying composite risk models to personalize diagnostic pathways—for instance, by adjusting the urgency or extent of biopsy based on a patient’s overall risk profile rather than PSA levels alone. Finally, this approach enables clinicians to provide more nuanced patient counseling by explaining how each variable informs the decision-making process, thereby enhancing shared decision-making and potentially reducing overdiagnosis and unnecessary interventions. An apparent inconsistency was observed in that the multivariate logistic regression model achieved a slightly lower AUC (0.751) than PSA alone (0.782). This outcome does not necessarily indicate an analytical error, but rather reflects the fact that multivariate models do not always exceed the performance of the strongest single predictor. In our dataset, PSA demonstrated particularly strong discriminative power, while the additional covariates (prostate volume, PI-RADS score, and age) contributed only modestly and may have introduced statistical noise. This effect is more pronounced in smaller cohorts, where model stability is limited, and has been described in prior methodological studies.

The combined biopsy approach achieved an 88.4% detection rate and outperformed either technique alone. This diagnostic gain demonstrates improved sensitivity for clinically significant prostate cancer; however, it also raises potential concerns about overdiagnosis of indolent lesions. Most additional detections involved Gleason 6 or 7 (3+4) tumors, which clinicians generally consider clinically significant, yet the balance between enhanced detection and the risk of overtreatment requires careful evaluation. These findings reinforce the need to refine patient selection criteria to optimize the clinical utility of combined biopsy strategies.

As reported in the literature, atypical small acinar proliferation (ASAP), which is commonly referred to as ATYP, carries a 40–50% likelihood of prostate cancer on repeat biopsy [15]. In our cohort, fusion-guided and systematic randomized biopsy demonstrated clear diagnostic interplay in patients with ATYP. Fusion-guided biopsy identified ATYP in 13 cases, and systematic biopsy subsequently confirmed prostate cancer (Gleason scores 6 or 7) in 10 of these patients (76.9%). Conversely, randomized biopsy detected ATYP in 10 patients, and fusion-guided biopsy confirmed malignancy in all of them. Only one patient demonstrated ATYP on both modalities. This reciprocal detection pattern underscores the diagnostic complementarity of the two techniques. Our findings provide compelling evidence that combining fusion and systematic biopsy substantially increases the likelihood of detecting clinically significant cancer in patients with ambiguous histopathological findings and support the conclusion that an integrative biopsy approach yields superior diagnostic accuracy.

To support the generalizability and clinical applicability of this study, we analyzed the cohort across key demographic and clinical parameters. This study enrolled men spanning a broad age range with varied PSA levels, prostate volumes, and PI-RADS scores, reflecting the diversity encountered in real-world practice. This variability allowed evaluation of biopsy performance and risk prediction models across different risk strata, enhancing the robustness of the findings and supporting the model’s applicability across heterogeneous clinical presentations, thereby increasing its diagnostic utility in routine practice.

Beyond clinical and imaging parameters, emerging evidence highlights how lifestyle, environmental, and genetic factors influence prostate cancer (PCa) risk. For example, a study in an Algerian population linked high consumption of red meat, animal fats, and dairy products to increased PCa incidence, whereas plant-based diets appeared protective [16]. A family history of PCa also increases risk, underscoring the disease’s multifactorial nature. A recent systematic review identified additional associations between PCa and long-term drug use (e.g., metformin, aspirin, and statins), metabolic disorders such as diabetes and obesity, and dietary patterns [17]. Although some associations remain inconclusive, these interrelated factors likely shape PCa risk cumulatively rather than independently. Future research should disentangle these complex relationships to refine personalized risk stratification models.

These findings highlight the complementary nature of fusion and randomized biopsy approaches, reinforcing the clinical value of using both methods to reduce the risk of false negatives and improve diagnostic accuracy in patients with suspicious or borderline histological findings.

In the specialty literature, the mp-MRI seemed to upgrade 18% of the patients from the low-risk group to the intermediate- or high-risk groups [18]. Another study conducted on 100 patients undergoing 14-core TRUS-guided biopsy and mp-MRI-TRUS fusion showed an increase in PC detection rate, from 56% in the single technique to 62% in fusion [11].

The presence of false-negative guided biopsy is not uncommon, yet concerns of missed disease often remain, especially when the mp-MRI shows a possible lesion. There can also be false-positive results on mp-MRI, such as “PCa mimics” [14]. Rosenkrantz, A.B. et al. highlighted 10 pitfalls that may occur while interpreting an mp-MRI. This includes normal anatomic structures that can be mistaken for a tumor (such as the central zone with decreased T2 signal intensity and decreased ADC) or post-inflammatory scars [19].

The comparison between conventional 12-core TRUS-guided biopsy and mp-MRI-fusion-guided biopsy has received considerable attention in recent years. Numerous studies, systematic reviews, and meta-analyses have evaluated these techniques, with growing evidence suggesting that fusion-guided biopsy offers superior prostate cancer detection rates, particularly for clinically significant tumors [20,21,22,23,24]. This trend has been statistically validated across diverse patient populations and clinical settings. However, the outcomes of fusion biopsy are not universally consistent and can be influenced by several operator- and technology-dependent variables as well as the number of patients included in the study. For instance, the experience level of the urologist, particularly in performing and interpreting mp-MRI and fusion guidance, plays a crucial role in targeting accuracy. Similarly, the quality and performance of the ultrasound system used for image fusion can significantly impact lesion visualization and needle guidance precision. In our study, these variables may have played a role in the lack of statistically significant differences observed between fusion and randomized biopsy techniques, despite a clear trend toward higher overall detection when both methods were used in combination.

### 4.2. Future Directions

The results of this study could inform the future development of a follow-up protocol utilizing 68Ga-PSMA PET-CT imaging to enhance the detection of local recurrences and metastatic disease, as well as monitor long-term survival outcomes in prostate cancer patients. Additionally, 68Ga-PSMA PET-CT may offer an opportunity to refine and personalize biopsy strategies, particularly in patients presenting with elevated PSA levels and/or highly suspicious imaging findings. By integrating advanced molecular imaging into diagnostic and surveillance pathways, a more targeted and individualized approach to prostate cancer management could be achieved, potentially improving both diagnostic accuracy and patient outcomes. Given the limitations of conventional imaging in accurately staging intermediate- and high-risk prostate cancer, researchers should incorporate 68Ga-PSMA PET-CT to refine preoperative assessment. Moreover, integrating PSMA-based imaging into future diagnostic algorithms could improve lesion characterization and guide targeted interventions, as recent studies suggest [25].

Although advances in detection and treatment have markedly improved prostate cancer survival [26,27,28], researchers must now focus on survivorship issues. Clinicians should address health-related quality of life and psychological well-being, including depression, while recognizing that some patients may require individualized psychosocial support [3,29]. Emerging evidence also indicates that long-term survivors, especially those with a family history of cancer, face increased risk of second primary malignancies [30]. Therefore, future studies should incorporate these factors to develop personalized follow-up strategies for prostate cancer survivors.

### 4.3. Other Observations

Prostatic amyloidosis is typically an incidental histopathological finding that is often discovered during biopsies performed for other indications. A retrospective study conducted within a single healthcare unit identified only 40 cases of prostatic amyloid deposits over a 21-year period (2001–2022), highlighting its rarity [31]. In our cohort, we similarly observed one incidental case of prostatic amyloidosis, further emphasizing the uncommon nature of this finding.

Granulomatous prostatitis (GP) is a rare benign inflammatory condition of the prostate that can closely mimic prostate cancer both clinically and radiologically, often presenting with elevated PSA and suspicious mp-MRI findings. Autoimmune mechanisms, as highlighted in recent reports such as cases occurring in patients with psoriatic arthritis, further complicate its recognition and underscore its potential to confound cancer diagnostics [32]. In our cohort, we specifically examined histopathological outcomes for features suggestive of GP, given its relevance as a prostate cancer mimic; however, no such cases were identified. Nevertheless, awareness of GP and other inflammatory conditions remains essential, as their presence could significantly impact the diagnostic accuracy of combined biopsy strategies and the interpretation of mp-MRI findings.

### 4.4. Limitations

This study has several limitations that should be acknowledged. First, the retrospective design may introduce inherent selection and information biases, despite efforts to apply consistent inclusion and exclusion criteria. Second, this study was conducted at a single center, which may limit the generalizability of the findings to broader populations or different clinical settings. Third, variability in operator experience and differences in ultrasound equipment performance may have influenced the accuracy of the fusion-guided biopsies, potentially impacting the comparison between the techniques.

Another important limitation relates to the unexpectedly low diagnostic performance of PI-RADS v2.1 observed in our study (AUC = 0.619), which contrasts with values consistently reported in large meta-analyses (AUC ≈ 0.86–0.89). This discrepancy likely reflects several study-specific factors. First, our database was restricted to men with relatively low PSA values (<25 ng/mL); this selection inherently reduces the contrast between malignant and benign cases and thus lowers discriminative accuracy. Second, radiologists had access to PSA levels at the time of image interpretation, which may have introduced an unconscious bias toward more conservative PI-RADS scoring in the context of low PSA, thereby attenuating sensitivity. Third, our single-center design and modest sample size (n = 138) limit the stability of AUC estimates compared with multicenter meta-analyses. Finally, by only including patients with PI-RADS ≥ 3, we altered the case mix and excluded very low-risk lesions, which may have further distorted the apparent predictive performance of PI-RADS when considered in isolation. Taken together, these factors suggest that our PI-RADS AUC should not be interpreted as representative of its general diagnostic utility, but rather as a context-specific finding shaped by the characteristics of our cohort and methodology. We did not assess interobserver variability in PI-RADS scoring, which could affect the consistency and reproducibility of imaging-based risk stratification.

Additionally, lifestyle factors such as diet, physical activity, and medication history, which can influence prostate cancer risk, were not included, potentially limiting the comprehensiveness of the risk model. Future prospective multicenter studies with standardized biopsy protocols and longer follow-up are required to validate and expand upon these findings.

## 5. Conclusions

This study highlights the complementary roles of fusion-guided and systematic randomized prostate biopsies in improving prostate cancer detection rates. While McNemar’s test demonstrated no statistically significant difference between fusion and randomized biopsy when considered independently, the combined approach significantly outperformed each technique used in isolation, underscoring the added diagnostic value of integrating both strategies. These findings suggest that relying on a single biopsy method risks underdiagnosis, whereas a combined approach maximizes the detection of clinically significant cancers.

Additionally, the multivariate analysis confirmed that clinical parameters such as PSA levels, prostate volume, age, and PI-RADS score each contributed independently to cancer prediction, with PSA demonstrating the highest individual discriminative power. The absence of significant multicollinearity among predictors reinforces the robustness and interpretability of the multivariate model.

Given the increasing survival rates and evolving patient profiles, future research from our group aims to further investigate risk stratification strategies, familial cancer predisposition, and the potential role of novel imaging techniques such as 68Ga-PSMA PET-CT in improving post-biopsy surveillance. Personalized biopsy strategies tailored to patient-specific risk factors may offer the next step forward in optimizing prostate cancer detection and management.

## Figures and Tables

**Table 1:** Number of patients with positive prostate cancer biopsy results according to the technique used.

Technique	Positive Cases
Fusion positive	94
Randomized positive	105
Fusion positive, randomized negative (F+/R−)	16
Randomized biopsy (F−/R+)	28
Both positive (F+ ∩ R+)	78
Positive via any method (F+ ∪ R+)	122
Fusion negative, randomized negative (F−/R−)	16
Total patients	138

+ positive, − negative.

**Table 2:** Contingency tables comparing the detection performance of fusion and randomized prostate biopsies versus the combined approach (fusion + randomized).

Fusion vs. Combined		
	Combined+	Combined−
Fusion+	94	0
Fusion−	28	16
Randomized vs. Combined		
	Combined+	Combined−
Randomized+	105	0
Randomized−	17	16

+ positive, − negative.

**Table 3:** Multicollinearity diagnostics for the predictor variables used in the regression model. Tolerance and Variance Inflation Factor (VIF) values confirm the absence of significant multicollinearity, indicating that each variable contributes independently to the model.

Parameter	Tolerance	VIF
AGE	0.907	1.102
PSA	0.877	1.140
PI-RADS	0.873	1.146
PROSTATIC VOLUME	0.782	1.278

## Data Availability

All data are available from the corresponding author upon reasonable request.

## Abbreviations

The following abbreviations are used in this manuscript:

ATYP	Atypical glands suspicious for cancer
ADC	Apparent diffusion coefficient
BPH	Benign prostate hyperplasia
DCE	Dynamic contrast-enhanced
HGPIN	High-grade prostatic intraepithelial neoplasia
mp-MRI	Multiparametric magnetic resonance imaging
PCa	Prostate cancer
PI-RADS	Prostate Imaging Reporting and Data System
PSA	Prostate-specific antigen
T2W	T2-weighted
VIF	Variance inflation factor
